# The effects of obesity, diabetes and metabolic syndrome on the hydrolytic enzymes of the endocannabinoid system in animal and human adipocytes

**DOI:** 10.1186/1476-511X-13-43

**Published:** 2014-03-04

**Authors:** Jemma C Cable, Garry D Tan, Stephen PH Alexander, Saoirse E O'Sullivan

**Affiliations:** 1 Division of Medical Sciences & Graduate Entry Medicine, School of Medicine, Faculty of Medicine & Health Sciences, University of Nottingham, Royal Derby Hospital Centre, Uttoxeter Road, Derby, DE22 3DT, UK; 2 NIHR Biomedical Research Centre, Oxford University Hospitals NHS Trust, OCDEM, Churchill Hospital, Oxford, UK; 3 School of Life Sciences, University of Nottingham Medical School, Nottingham, NG7 2UH, UK

**Keywords:** Endocannabinoid, Adipose, Adipocyte, Obesity, Type 2 diabetes, Fatty acid amide hydrolase (FAAH), Monoacylglycerol lipase (MGL), Rat, Human

## Abstract

**Background:**

Circulating endocannabinoid levels are increased in obesity and diabetes. We have shown that fatty acid amide hydrolase (FAAH, an endocannabinoid hydrolysing enzyme) in subcutaneous adipose tissue positively correlates with BMI in healthy volunteers. The aim of the present study was to investigate whether the hydrolytic enzymes of the endocannabinoid system are affected by diabetes or metabolic syndrome in obesity.

**Methods:**

Using radiolabelled substrates, FAAH and monoacylglycerol lipase (MGL) activities were assessed in adipocytes from various adipose depots in Zucker rats (n = 22, subcutaneous abdominal, visceral and epididymal) and bariatric patients (n = 28, subcutaneous abdominal and omental).

**Results:**

FAAH activity was significantly increased in adipocytes of obese (Zucker Fatty) compared to Zucker lean rats (P < 0.05) but was not raised in the Zucker Diabetic Fatty rats (ZDF). MGL activity was raised in both Zucker Fatty (P < 0.001-0.01) and ZDF rats (P < 0.05) and was positively correlated with body weight and plasma glucose levels (P < 0.01). In bariatric patients (BMI range 37–58 kg.m^2^), there was a trend for MGL activity to correlate positively with BMI, reaching significance when type 2 diabetic patients were removed. FAAH and MGL activities in obese humans were not correlated with blood pressure, skinfold thicknesses, fasting glucose, insulin, HbA1c, triglycerides or cholesterol levels.

**Conclusions:**

FAAH in adipocytes is differentially altered in animal models of obesity and diabetes, while MGL activity is increased by both. However, in obese humans, FAAH or MGL activity in adipocytes is not affected by diabetes, dyslipidaemia or other markers of metabolic dysfunction. This suggests increased circulating levels of endocannabinoids are not a result of altered degradation in adipose tissue.

## Introduction

The endocannabinoid system (ECS) is crucial in the regulation of metabolism and energy homeostasis (see [1-4]). The ECS is comprised of endocannabinoid ligands, their receptors, and the enzymes required for their synthesis and degradation. *N*-arachidonoylethanolamide (anandamide, AEA) [5] and 2-arachidonoylglycerol (2-AG) [6,7] are the two best characterised endocannabinoids, and the enzymes which degrade them are predominantly fatty acid amide hydrolase (FAAH) and monoacylglycerol lipase (MGL) respectively [8,9]. Other N-acylethanolamines such as oleoylethanolamide (OEA) and palmitoylethanolamide (PEA) are also degraded by FAAH.

It has been suggested that the ECS is upregulated in human obesity on the basis that plasma concentrations of AEA [10,11], 2-AG [12] and other acyl-ethanolamides [13] correlate positively with body mass index (BMI). We have shown that FAAH activity in mature subcutaneous adipocytes correlates positively with BMI in healthy volunteers [14]. However, BMI is a crude measure of adiposity and the adverse metabolic consequences of obesity are more closely related to centripetal obesity. It is therefore relevant that circulating 2-AG levels [10] and FAAH activity in subcutaneous adipocytes [14] also correlate with waist circumference, and the most significant rise in plasma 2-AG occurs in those with visceral obesity [11,12].

The peripheral ECS may be dysregulated in type 2 diabetes, as first suggested in a study showing increased concentrations of AEA and 2-AG in plasma in patients with type 2 diabetes compared to BMI-matched controls [15]. In subcutaneous adipose tissue, AEA is increased and 2-AG is decreased in obese humans with type 2 diabetes compared to lean and obese non-diabetic controls [16]. Hyperinsulinaemia (using a clamp) also causes an upregulation of FAAH mRNA in subcutaneous abdominal adipose tissue of lean subjects, but no change in the obese group, in which FAAH was already chronically upregulated [17]. However, any influence of hyperinsulinaemia or other metabolic factors on functional FAAH or MGL enzyme activity (as opposed to mRNA levels) have not been assessed.

Visceral adipose tissue is more metabolically active than subcutaneous adipose tissue [18], but there is conflicting evidence as to whether the ECS differs significantly between these depots. In subjects with a BMI less than 25 kg.m^2^, cannabinoid 1 (CB_1_) receptor mRNA expression is higher [19,20], unchanged [21] or lower [11] in subcutaneous compared to visceral adipose tissue. In obese subjects, CB_1_ receptor mRNA expression is elevated [11,19,21] or not different [20] in visceral compared to subcutaneous adipose tissue. FAAH mRNA expression is increased [11] or unchanged [21] in visceral compared to subcutaneous adipose tissue. A higher expression of MGL mRNA in subcutaneous compared to visceral adipose [21] is consistent with increased levels of 2-AG reported in visceral adipose tissue [15]. However, the catalytic activities of FAAH and MGL have not been compared between different adipose tissue depots.

In light of this background, the primary aim of the current study was to investigate if obesity co-morbidities and metabolic risk factors, including hyperinsulinaemia, hyperglycaemia and dyslipidaemia, influence FAAH and MGL enzyme activities in adipose tissue. The secondary aim was to determine whether FAAH or MGL activities differ between visceral and subcutaneous adipocytes. These objectives were first explored in rat models of obesity and type 2 diabetes, and subsequently in severely obese patients undergoing bariatric surgery.

## Results

### Enzyme activity

**Figure 1:**
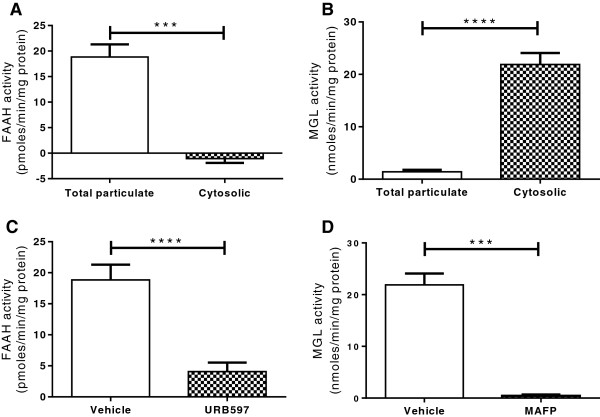
**Endocannabinoid degradation enzyme assay validation.** FAAH activity was detected in the total particulate and not cytosolic fraction of adipocyte homogenates of Zucker rats (**A**, n = 48), and was significantly inhibited by URB597 (1 μM, **C**, n = 48). MGL activity was detected in the cytosolic and not total particulate fraction of adipocyte homogenates (**B**, n = 33), and was significantly inhibited by MAFP (1 μM, **D**, n = 66). Data are given as means with error bars representing S.E.M and were analysed by a Mann–Whitney test (*** *P* < 0.001, *** *P* < 0.001).

In the rat samples tested, as expected, FAAH activity was present in the total particulate fraction of the homogenised adipocytes, but was not detected in the cytosolic fraction, while the majority of adipocyte MGL activity was detected in the cytosolic fraction (Figure 1). Anandamide hydrolysis was suppressed by the FAAH inhibitor URB597 (P < 0.0001) and 2-OG hydrolysis was suppressed by a non-specific MGL inhibitor, methoxy arachidonyl fluorophosphonate (MAFP, P < 0.0001, Figure 1). The % coefficient variation of FAAH and MGL assays performed in duplicate were 15.96 ± 15.13 (mean ± SD) and 12.88 ± 15.13 respectively.

### The effects of obesity/diabetes on FAAH and MGL activity in Zucker rats

**Figure 2:**
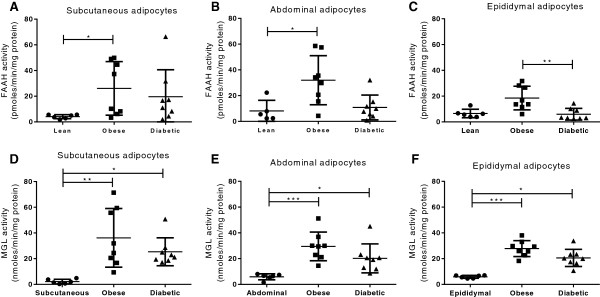
**Endocannabinoid degradation enzymes in lean and obese Zucker rats.** FAAH and MGL activities in mature adipocytes isolated from subcutaneous **(A,D)**, abdominal **(B,E)** and epididymal **(C,F)** adipose tissue depots in lean (*n* = 6), obese (*n* = 8) and obese diabetic Zucker rats (*n* = 8). Data are given as means, with error bars representing S.D., and were analysed by Krustal Wallis with comparisons between the means of all data (* *P* < 0.05, ** *P* < 0.01, *** *P* < 0.001).

**Figure 3:**
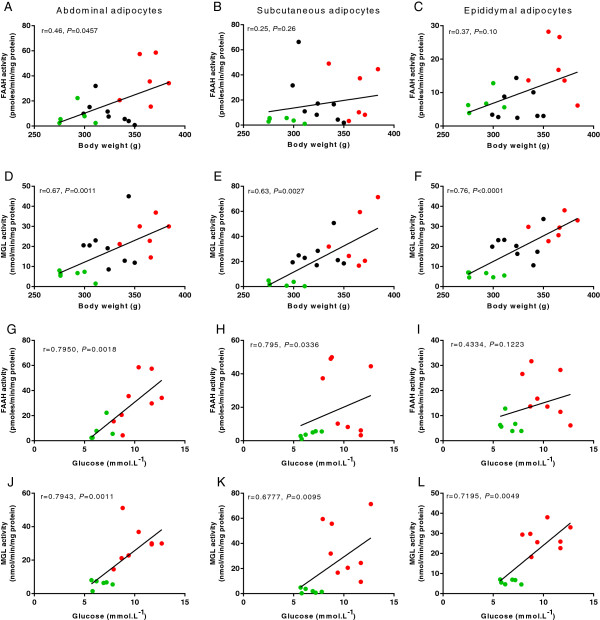
**Correlation studies in Zucker rats.** Correlative studies between body weight and enzyme activity in mature adipocytes isolated from each adipose tissue depot in abdominal **(A,D)**, subcutaneous **(B,E)**, and epididymal **(C,F)** adipocytes. When the Zucker diabetic rats were omitted from analysis, a significant correlation was observed between FAAH activity and plasma glucose levels in the abdominal **(G)** and subcutaneous **(H)** but not epididymal adipocytes **(I)**. Stronger correlations were also seen between MGL activity and plasma glucose levels in all adipose depots **(J,K,L)**. The Spearman correlation coefficient is reported. Key: green, lean rats; red, obese rats; black, obese diabetic rats.

Comparing FAAH activity in adipose depots from obese diabetic rats with that from lean and obese Zuckers indicated levels of activity more similar to those observed in lean Zuckers than in obese (Figure 2A-C). In fact, FAAH activities in adipocytes from obese diabetic rats were significantly reduced compared to those from obese animals in epididymal adipocytes (P < 0.01, Figure 2C). Within all rat strains, abdominal adipocyte FAAH activity correlated positively with body mass (P < 0.05, Figure 3A). There was no correlation between blood glucose levels and FAAH activity (data not shown); however, when the diabetic animals were removed from this analysis, a positive correlation was observed between FAAH activity and blood glucose in the subcutaneous and abdominal adipocytes (see Figure 3G,H).

In subcutaneous adipocytes, MGL activities for the obese and obese diabetic rats were 18- and 12-fold increased compared to the lean animals (*P* < 0.01 and *P* < 0.05, Figure 2D). In abdominal adipocytes the corresponding values were 5- and 3-fold, respectively (*P* < 0.001 and *P* < 0.05, Figure 2E), and in epididymal adipocytes, MGL activity was 5- and 3.5-fold increased (*P* < 0.001 and *P* < 0.05, Figure 2F). A positive relationship was identified between MGL activity and body mass in all adipose tissues (abdominal r = 0.67, P < 0.001; subcutaneous r = 0.63, P < 0.01; epididymal r = 0.76, P < 0.0001, Figure 3D,E,F). MGL activity also correlated with blood glucose levels in adipocytes from all three adipose tissue depots (subcutaneous r = 0.53, P < 0.05; abdominal r = 0.56, P < 0.01; epididymal r = 0.48, P < 0.05), and this effect became stronger when the diabetic rats were removed (See Figure 3J,K,L).

### The effects of adipose depots on FAAH and MGL activity

**Figure 4:**
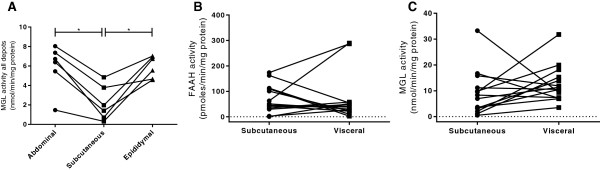
**Endocannabinoid degradation enzymes in different adipose depots.** MGL activity in paired samples of adipose tissue from lean Zucker rats **(A)**. FAAH **(B)** and MGL **(C)** activities in paired samples of subcutaneous and visceral mature adipocytes from obese humans (*n* = 14). Data are presented as scatterplots and were analysed using Krustal Wallis with comparisons between the means of all data **(A)** or by Wilcoxon matched-pairs signed rank test (**B** and **C**, *P < 0.05).

In the lean rats, there was no significant difference in FAAH activity between adipose depots (see Figure 2A-C), but MGL activity was found to be lower in the subcutaneous adipocytes than either the abdominal and epididymal adipocytes (*P* < 0.05, Figure 4A). In the obese and obese diabetic rats, neither FAAH nor MGL activity in adipocytes differed between the three adipose tissue depots tested (see Figure 2A-F). Similarly, in human adipocytes, FAAH (Figure 4B) and MGL (Figure 4C) activities did not differ between paired subcutaneous and visceral adipocytes. All subsequent analysis was carried out in subcutaneous adipocytes.

### FAAH and MGL activity in obese patients

The physiological characteristics of these severely obese patients are given in Table 1, which shows the sample divided into three groups: patients with clinically diagnosed T2M (n = 10), patients without T2M but with at least three markers of metabolic syndrome (21, n = 12), and patients without diabetes and with only one or two markers of metabolic syndrome (‘healthy’, n = 6). Between these groups, age, BMI, fasting serum insulin concentration, HOMA and mean arterial pressure did not differ. The mean fasting serum glucose concentration and HbA1c were higher in the diabetic group than both the healthy and metabolic syndrome groups (*P* < 0.05). Patients in all groups were prescribed similar medications for dyslipidaemia and hypertension, but 5 patients in the diabetic group were taking hypoglycaemic medication compared to none in the healthy and metabolic syndrome groups.

**Table 1:** Characteristics of the patients included in this study

	**‘Healthy’ (*n*** **= 6)**	**Metabolic syndrome (*n*** **= 12)**	**Diagnosed T2D (*n*** **= 10)**
**Gender split (male: female)**	0:6	3:8	2:8
**Age (years)**	44.0 ± 4.7	44.5 ± 3.3	45.5 ± 2.7
**BMI (kg.m**^**-2**^**)**	44.0 ± 4.7	46.3 ± 1.7	44.8 ± 1.5
**Insulin (mU.L**^**-1**^**)**	12.2 ± 3.1	14.0 ± 2.3	15.9 ± 1.9
**Glucose (mmol.L**^**-1**^**)**	5.0 ± 0.2^~^	5.1 ± 0.2*	8.7 ± 1.4^~^*
**HbA1c (%)**	5.8 ± 0.1^†^	5.8 ± 0.3^#^	7.9 ± 0.7^†#^
**HOMA2-%S**	85.8 ± 26.2	71.4 ± 14.2	50.4 ± 6.8
**MAP (mmHg)**	96.1 ± 4.8	96.3 ± 4.3	104.8 ± 3.6
**Dyslipidaemia medication**	1 (statin)	1 (statin)	2 (statin)
**Hyperglycaemia medication**	0	0	5 (metformin)
**Hypertension medication**	3 (1: ACE inhibitor; 2: ACE inhibitor + thiazide diuretic)	3 (1: ACE inhibitor; 1: ACE inhibitor + β_1_ antagonist; 1: ACE inhibitor + thiazide diuretic)	3 (2: ACE inhibitor; 1: thiazide diuretic)

Values are given as mean ± S.E.M. ^~^*^†#^Values marked with the same characters are significantly different from each other (P < 0.05). Data analysed using ANOVA and Bonferroni *post* hoc test.

**Figure 5:**
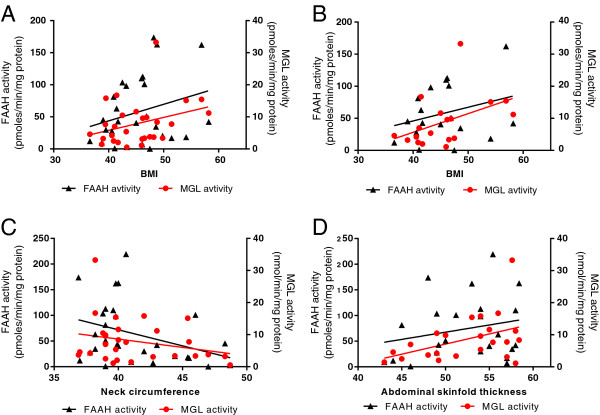
**Endocannabinoid degradation enzymes in obese humans.** The relationship between FAAH and MGL activity and BMI in the whole population **(A)** and in the non-diabetic patients **(B)**, and between FAAH and MGL activity and neck circumference **(C)** and abdominal skinfold thickness in the female only population **(D)**.

Within this sample of obese patients, neither FAAH activity nor MGL activity in subcutaneous adipocytes correlated with BMI or waist circumference, although there was a trend for a positive correlation between MGL activity and BMI (*P* = 0.0975, r = 0.3195, Figure 5A). Removing patients with diabetes revealed a significant correlation between MGL and BMI (*P* = 0.0408, r = 0.4861, Figure 5B). FAAH and MGL activity were not correlated with various skinfold measurements (abdominal, tricep, bicep, arm, iliac crest, subscapular, and axilla). There was a trend for a negative correlation between FAAH activity and neck circumference (*P* = 0.061, r = -0.3586, Figure 5C). In the female only population, a positive correlation was seen between MGL activity and abdominal skin fold thickness (*P* = 0.0406, r = 0.43, Figure 5D).

**Figure 6:**
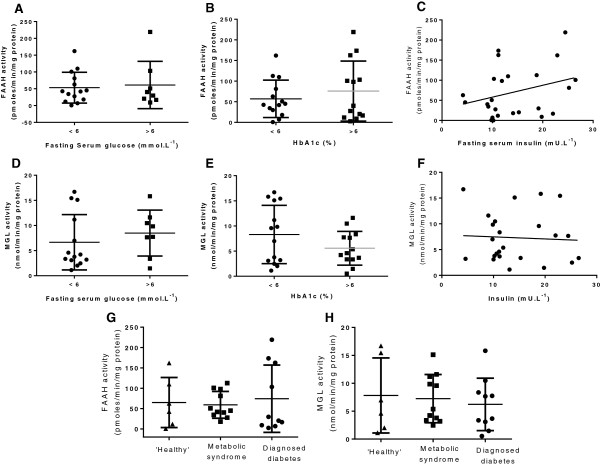
**Endocannabinoid degradation enzymes in metabolic syndrome and diabetes.** The effects of fasting serum glucose concentration **(A,D)**, HbA1c **(B,E)** and insulin concentration **(C,F)** on FAAH and MGL activities in subcutaneous adipocytes from obese humans. Data are presented as scatterplots, with error bars representing S.D., and were analysed using Student’s *t* test or linear regression. FAAH **(G)** and MGL **(H)** activities in subcutaneous adipocytes from obese humans assigned to one of three groups based on the following criteria: healthy <2 components of metabolic syndrome (*n* = 6, triangles); metabolic syndrome ≥3 components of metabolic syndrome (*n* = 11, squares); diagnosed type 2 diabetes with or without metabolic syndrome (*n* = 10, circles). Data are presented as scatterplots, with error bars representing S.D., and were analysed using one way ANOVA and Bonferroni *post* hoc test.

**Figure 7:**
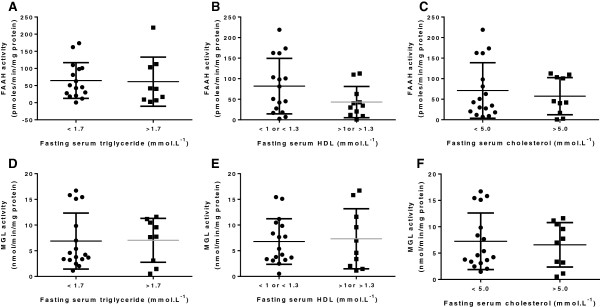
**Endocannabinoid degradation enzymes and blood lipids.** The effects of fasting serum triglyceride concentration **(A,D)**, total cholesterol concentration **(C,F)**, and HDL cholesterol concentration **(B,E)** on FAAH and MGL activities in subcutaneous adipocytes from obese humans. Data are presented as scatterplots, with error bars representing S.D., and were analysed using Student’s *t* test.

FAAH and MGL activity were not different between ‘healthy’ patients and those with metabolic syndrome or diagnosed T2M (Figure 6G,H). FAAH and MGL activity did not correlate with blood pressure, heart rate or age (data not shown), and were not different in patients with a normal compared to high fasting glucose, or HbA1c and were not correlated with plasma insulin levels (Figure 6). FAAH or MGL activity in subcutaneous adipocytes was also not different between high and normal risk groups with regard to triglycerides, total cholesterol or HDL-cholesterol (see Figure 7). There was also no correlation between FAAH or MGL activity with non-HDL-C (data not shown).

## Discussion

The primary aim of this study was to determine whether the activities of FAAH and MGL, two key catabolic enzymes of the ECS, are differentially affected by diabetes or other markers of the metabolic syndrome in obesity. FAAH was raised in obese rats, but not obese diabetic rats, while MGL activity was elevated in both strains. FAAH and MGL activities positively correlated with body weight and blood glucose in the Zucker rats, but MGL activity correlated more strongly. By contrast, in severely obese humans, FAAH and MGL activity in adipose tissue was not correlated with adiposity and were not different between ‘healthy’, type 2 diabetic, metabolic syndrome patients, or in patients with clinical elevated blood glucose, poor glycaemic control or hyperlipidaemia. FAAH and/or MGL activities were not different between visceral and subcutaneous adipose tissue depots, except in the lean rats, where MGL activity was higher in visceral compared to subcutaneous adipocytes.

### The effects of obesity/diabetes on FAAH and MGL activity in Zucker rats

We found that FAAH and MGL enzyme activities in mature adipocytes are increased in Zucker rats compared to lean rats, and were positively correlated with body mass in the rat strains MGL activity was also correlated with blood glucose levels, and this relationship was stronger when the diabetic rats were removed from analysis. A relationship between FAAH and blood glucose was also observed without the diabetic rats. Given that MGL has distinct roles in lipolysis and signalling, the increased activity of MGL in our studies may reflect an increase in the endocannabinoid signalling role of MGL rather than lipolysis, since lipolytic enzymes are generally repressed in obesity. MGL activity was raised in both the obese and obese diabetic rats suggesting a differential regulation of FAAH and MGL in diabetes in obesity. The physiological consequence of this differential effect of diabetes in obesity on enzyme activity is not clear. In obese diabetic humans, AEA, but not 2-AG, is increased in subcutaneous adipose tissue [16]. An increase in AEA causing activation of CB_1_ could lead to unfavourable metabolic effects, however an increase in PEA and OEA, which are also degraded by FAAH [16], may be beneficial. For example, OEA decreases food intake and lowers hyperlipidaemia in obese rats via PPARα activation [22] and both OEA and PEA can stimulate insulin and GLP-1 secretion via activation of GPR119 [23,24].

### The effects of metabolic markers on FAAH and MGL activity in obese patients

Although we have shown in a healthy volunteer population that FAAH enzyme activity in mature subcutaneous adipocytes correlates with BMI (ranging from 19.1-33.8 kg.m^-2^, 13), in the current population of severely obese patients (BMIs 36.6 to 58.2 kg.m^-2^), there was no further correlation between FAAH activity and BMI, waist circumference, neck circumference or skinfold thicknesses. Interestingly, a 5% reduction in total body weight following calorie restriction does not affect FAAH mRNA [10] and a 10% weight loss in obese volunteers resulted in a decrease in FAAH mRNA in gluteal, but not abdominal, adipose tissue to levels lower than the lean controls [25]. A possible conclusion from these findings is that there is limit to the enzymatic capacity of FAAH which no longer increases in proportion to BMI in our severely obese patients.

In contrast to the data with FAAH activity, and in accordance to the animal data, we found a trend for a positive correlation in the human bariatric patients between MGL and BMI (becoming significant without diabetic patients) and between MGL and adiposity in the female only population. This confirms the differential regulation of FAAH and MGL activity in adipocytes. Since FAAH is regulated by both insulin and leptin, we hypothesise that factors such as insulin and leptin resistance in obesity oppose any further increases in FAAH activity and that MGL must be under other regulating factors.

In obese patients, there was no difference in FAAH or MGL between patients with or without a diagnosis of type 2 diabetes, or those with clinically elevated plasma glucose, HbA1c or HOMA. Furthermore, no correlation was observed between serum insulin levels and FAAH or MGL activity. Together this suggests that within a severely obese population, these metabolic variables do not appear to influence FAAH or MGL activity. Similarly, elevated blood pressure, neck circumference, triglycerides, total cholesterol or HDL levels did not correlate with FAAH or MGL activity. However, it should be noted that our patients achieved reasonably good glycaemic control (HbA1c 7.9 ± 0.7), and were not hyperinsulinaemic. Therefore, the finding that FAAH activity was reduced in the ZDF rat and not diabetic humans might be ascribed to the fact that diabetes is uncontrolled in the ZDF, and this idea should be further pursued. Interestingly, another team reported that FAAH mRNA in subcutaneous adipose tissue correlated positively with blood glucose and insulin in men, but not in females [11], which may be important given that the majority of the patients in the current study were female. Indeed, we observed a significantly lower level of FAAH in our obese males. Any gender differences in the regulation of FAAH might explain why our predominantly female population did not show any significant relationship between FAAH activity and variables such as insulin sensitivity.

### FAAH and MGL activities in different adipose depots

Visceral adipose tissue is thought to have greater metabolic activity and a greater impact on cardiovascular health [18]. Differences between adipose depots have been reported for various components of the ECS. In obese humans, CB_1_ receptor mRNA is higher in visceral adipose tissue than subcutaneous [19,21]. FAAH mRNA levels are the same between subcutaneous and visceral adipose tissue in obese humans [1], or higher in visceral than subcutaneous/gluteal adipose tissue in lean and obese humans [11]. MGL is reported to be downregulated in visceral adipose tissue [21,25]. No studies have been published on the activities of FAAH or MGL enzymes in adipocytes from different adipose tissue depots. In the present study, we found that FAAH and MGL activities were not different in paired subcutaneous and visceral adipocytes from obese patients. Similarly, no differences were observed between the rat strains in enzyme activity between adipose depots tested. This suggests that the rate of endocannabinoid degradation does not differ between visceral and subcutaneous mature adipocytes and it may be that differences in the expression of the ECS in the stromal-vascular fraction may account for the overall differences in mRNA observed in other studies between depots, or that enzyme mRNA does not reflect enzyme activities.

## Conclusion

In summary, several previous studies have shown that in obese humans, circulating endocannabinoid levels and components of the ECS in adipose tissue are altered by insulin or diabetes. The results presented in this study show that FAAH and MGL enzyme activities are increased in adipocytes from animal model of diabetes/obesity. However, in subcutaneous mature adipocytes from severely obese humans, FAAH and MGL enzyme activities are not altered in relation to BMI, waist circumference, adipose tissue distribution, blood pressure, fasting glucose or insulin, glycaemic control or dyslipidaemia. Differences between the animal and human studies may be explained by gender, or differences in insulin (and potentially other hormones) sensitivity. No differences in the activity of FAAH or MGL were identified between subcutaneous and visceral adipocytes.

## Methods

This study was approved by Derbyshire Regional Ethics Committee (08/H0401/94) and Royal Derby Hospital Trust (DHRD/2008/081), and recruited patients undergoing elective laparoscopic bariatric or cholecystectomy surgery at Royal Derby Hospital. Informed written consent was obtained in accordance with Good Clinical Practice and the Declaration of Helsinki. The animals were used in accordance with the Home Office Code of Practice for the Housing and Care of Animals used in Scientific Procedures and were killed by an appropriate humane Schedule 1 method (cervical dislocation).

### Animal models

Three strains of male Zucker rat were used; the lean Zucker control (289 ± 6 g, 6.6 ± 0.3 mmol.L^-1^ glucose, n = 6), Zucker Fatty (obese, 363 ± 7 g, 10.2 ± 0.6 mmol.L^-1^, n = 8) and Zucker Diabetic Fatty (ZDF, obese diabetic, 325 ± 7 g, 18.3 ± 1.0 mmol.L^-1^, n = 8). After sacrifice, adipose tissue was immediately dissected from the subcutaneous abdominal (inguinal), visceral (perirenal) and epididymal adipose depots and immediately stored at -80°C. Blood glucose concentration was measured using a glucometer (Optium Xceed, Abbott Laboratories Ltd., UK).

### Clinical studies

Patients (23 women and 5 men, this split is representative of the fact that more women were undergoing bariatric surgery at the time) were recruited into one of three groups; those diagnosed with T2M (diabetic, n = 10); patients without diabetes, but with at least three markers of metabolic syndrome (metabolic syndrome, n = 12) [26]; and those without diabetes and with fewer than two markers of metabolic syndrome (‘healthy’, n = 6). Markers of metabolic syndrome included waist circumference ≥94 cm (male) or ≥80 cm (female); serum triglyceride ≥1.7 mmol.L^-1^; serum HDL-cholesterol <1 mmol.L^-1^ (male) or <1.3 mmol.L^-1^ (female); systolic blood pressure ≥130 mm Hg and/or diastolic blood pressure ≥85 mm Hg; and fasting serum glucose ≥5.6 mmol.L^-1^. Omental (n = 14) and subcutaneous adipose (n = 28) biopsies approximately 2 cm by 3 cm by 0.5 cm in size were taken atraumatically without heat coagulation. The samples were stored in ice-cold physiological salt solution before immediate transfer to the laboratory and stored within one hour at -80°C. Blood for serum glucose, insulin, triglycerides and cholesterol was processed and analysed routinely at the Royal Derby Hospital Pathology Laboratories.

Blood pressure was measured with subjects rested and supine. Anthropometric measurements performed by one trained person while the patient was standing. Waist circumference was measured at the midpoint between the iliac crest and costal margin, and hip circumference was taken at the widest point around the hips. Neck circumference was measured at the level of the cricothyroid cartilage and arm circumference was measured at the midpoint between the shoulder and elbow. Skinfold thickness was measured at 7 anatomical sites using Harpenden calipers. The 7 sites were: tricep (posterior, level with circumference), bicep (anterior, level with circumference), subscapular (parallel with inferior angle of scapular), suprailiac (immediately superior to iliac crest), abdominal (2 cm to the side of umbilicus), chest (as high as possible between anterior axillary line and nipple) and midaxillary (on midaxillary line, level of xiphoid process of sternum) [27].

### Isolation of mature adipocytes

The method used to obtain mature adipocytes was adapted from Rodbell [28] as we have previously published [14] for both human and rat adipose samples. Adipose samples were thawed on ice, added to an equal volume of type II collagenase in phosphate buffered saline (PBS, 1 mg.ml^-1^, Sigma-Aldrich, UK) and digested at 37°C for 45 minutes. The samples were washed twice in PBS using centrifugation (500 x *g*, 2 minutes) to separate the mature adipocytes which formed a floating layer. The isolated mature adipocytes were stored at -80°C until homogenisation. Isolated mature adipocytes were homogenised in TE buffer (50 mM Tris, 1 mM EDTA, pH 7.4) using a hand-held glass homogeniser on ice. The homogenates were centrifuged (18,000 x *g*, 10 minutes) and the supernatant removed and spun again (20,000 x *g*, 30 minutes). The supernatant layer from this step was then stored at -80°C as the cytosolic fraction. The cellular pellet was homogenised in PBS (10 mM phosphate, 2.7 mM potassium chloride, 137 mM sodium chloride, pH 7.4), centrifuged (20,000 x *g*, 30 minutes), resuspended and stored at -80°C as the total particulate fraction.

### Enzyme activity assays

Enzyme assays were carried out with minor modifications of the method of Boldrup *et al.*[29]. Samples of the total cell particulate or cytosolic fraction (in duplicate) were diluted in TE buffer (fatty acid free albumin 1 mg.ml^-1^, pH 7.4) and at 37°C for 10 min with the FAAH inhibitor URB597 (1 μM, Sigma Chemical Company, UK) or vehicle. [^3^H]-AEA (American Radiolabelled Chemicals, USA) was added to a final concentration of 2 μM, and the samples were incubated at 37°C for 30 min. Activated charcoal (400 μl, 10% w/v in 0.5 M HCl) was used to stop the reaction. After brief centrifugation at approximately 13 000 x g, an aliquot of each supernatant layer was taken for scintillation counting. Tubes without homogenate were run in parallel to establish blank values. The total cell particulate or cytosolic fraction was assayed for MGL activity using the same method as above, substituting a MGL inhibitor, methylarachidonylfluorophosphonate (MAFP, 1 μM, Sigma Chemical Company, UK), and 2-oleoyl-[^3^H]-glycerol (2-OG, American Radiolabelled Chemicals, USA) to a final concentration of 100 μM and incubated at 37°C for 15 min. Concentrations of substrate (3H-AEA and 3H-2OG) were used at sub-saturating levels in order to allow visualisation of any changes in either substrate affinity or maximal hydrolysis rate.

### Statistical analysis

GraphPad Prism software was used to analyse the data. A D’Agostino & Pearson omnibus normality test was carried out on all data and non-parametric analysis applied where appropriate. Where linear regression was used, the Pearson or Spearman correlation coefficient is reported. When three groups were compared, one-way analysis of variance (ANOVA) was used with Bonferoni multiple comparison (or Krustal Wallis for non-parametric data). For comparison of two groups, either paired or unpaired Student’s *t* test (or Wilcoxon signed rank test) was used as appropriate.

## Competing interests

The authors declare that they have no competing interests.

## Authors’ contribution

JC and SPA performed the experiments. Experiments were devised by GT and SOS. JC and SOS analysed the data. All authors contributed and approved the manuscript.
